# Prospective multicenter study to identify optimal target population for motorized spiral enteroscopy

**DOI:** 10.1038/s41598-024-64510-w

**Published:** 2024-07-22

**Authors:** Antonio Giordano, Luis Compañy, Miriam Alajarin-Cervera, Francisco Antonio Ruiz-Gómez, Pedro Luis Fernández-Gil, Noelia Alonso-Lázaro, Javier Sola-Vera, Miguel Urpi-Ferreruela, Marta Aicart-Ramos, Sofía Parejo-Carbonell, Josep Maria Dedeu-Cuscó, César Prieto-Frías, Cintia Bógalo-Romero, Juan Egea-Valenzuela, Cristina Carretero, Vicente Pons-Beltrán, Begoña González-Suárez

**Affiliations:** 1 Endoscopy Unit, Gastroenterology Department, Hospital Clínic de Barcelona, Universitat de Barcelona, Barcelona, Spain; 2 Digestive Endoscopy Unit, Digestive Disease Department, Hospital General Universitari Dr. Balmis de Alicante, Alicante, Spain; 3 Department of Digestive Diseases, University Clinic Hospital Virgen de La Arrixaca, Murcia, Spain; 4 Gastroenterology and Hepatology Department, Valdecilla Research Institute (IDIVAL), Marqués de Valdecilla University Hospital, Santander, Spain; 5 Gastrointestinal Endoscopy Unit, Digestive Disease Department, Hospital Universitari I Politècnic La Fe, Grupo Acreditado de Investigación en Endoscopia Digestiva, Instituto de Investigación Sanitaria La Fe (IISLaFe), Valencia, Spain; 6 Endoscopy Unit, Gastroenterology Department, Hospital General Universitario de Elche, Elche, Spain; 7 Gastroenterology and Hepatology Department, Hospital Universitario Ramón y Cajal, Madrid, Spain; 8 Endoscopy Unit, Gastroenterology Department, Hospital del Mar, Barcelona, Spain; 9 Department of Gastroenterology, Clínica Universidad de Navarra, Pamplona, Spain

**Keywords:** Gastroenterology, Small intestine

## Abstract

Motorized spiral enteroscopy (MSE) enhances small bowel exploration, but the optimal target population for this technique is unknown. We aimed to identify the target population for MSE by evaluating its efficacy and safety, as well as detecting predictors of efficacy. A prospective multicenter observational study was conducted at 9 tertiary hospitals in Spain, enrolling patients between June 2020–2022. Analyzed data included demographics, indications for the procedure, exploration time, depth of maximum insertion (DMI), technical success, diagnostic yield, interventional yield, and adverse events (AE) up to 14 days from enteroscopy. Patients with prior gastrointestinal surgery, unsuccessful balloon enteroscopy and small bowel strictures were analyzed. A total of 326 enteroscopies (66.6% oral route) were performed in 294 patients (55.1% males, 65 years ± 21). Prior abdominal surgery was present in 50% of procedures (13.5% gastrointestinal surgery). Lower DMI (162 vs 275 cm, *p* = 0.037) and diagnostic yield (47.7 vs 67.5%, *p* = 0.016) were observed in patients with prior gastrointestinal surgery. MSE showed 92.2% technical success and 56.9% diagnostic yield after unsuccessful balloon enteroscopy (n = 51). In suspected small bowel strictures (n = 49), the finding was confirmed in 23 procedures (46.9%). The total AE rate was 10.7% (1.8% classified as major events) with no differences related to prior gastrointestinal/abdominal surgery, unsuccessful enteroscopy, or suspected small bowel strictures. The study demonstrates that MSE has a lower diagnostic yield and DMI in patients with prior gastrointestinal surgery but is feasible after unsuccessful balloon-enteroscopy and in suspected small bowel strictures without safety concerns.

Motorized spiral enteroscopy (MSE) is a novel technique for small bowel (SB) exploration that utilizes a 168-cm-long flexible endoscope (PowerSpiral, Olympus, Japan) with a motorized spiral overtube. The enteroscope is advanced by the endoscopist through the rotational movement of the overtube controlled by pedals.

The initial feasibility trial conducted in 2019 demonstrated the characteristics of the MSE device, highlighting short exploration times and deep insertion depth as its main features^1^. Subsequent clinical trials have reported high technical success and diagnostic yield with a total adverse rate in line with SB endoscopy^2–6^. Although the novel rotational technique could initially raise safety concerns in patients with prior abdominal surgery, three studies showed that efficacy and safety were not significantly affected in this patient population, however, the evidence was limited by a short cohort of patients and heterogeneous criteria for the classification of abdominal surgeries^3,5,6^.

Furthermore, there is a lack of evidence regarding the use of MSE in specific clinical scenarios encountered in daily practice. Patients with suspected small bowel strictures, characterized by a narrowed and rigid area of the intestine resulting from chronic inflammatory damage or scarring, may pose challenges for successful enteroscope passage and potentially lead to severe complications.

Comparative studies have shown similar rates of diagnostic yield and technical success between MSE and single or double-balloon enteroscopy^7–9^. However, MSE shows clear superiority when the objective is total SB exploration^10^. Nevertheless, the potential of MSE as an alternative when other techniques fail requires further investigation, particularly in patients with prior unsuccessful balloon enteroscopy.

In light of these considerations, the present study aims to identify the specific population that would benefit most from MSE by evaluating its efficacy and safety across different clinical indications.

## Methods

### Study design

A multicenter prospective observational study was conducted to evaluate the efficacy and safety of motorized spiral enteroscopy (MSE) performed at 9 tertiary Hospitals in Spain from June 2020 to June 2022. The data analyzed are part of a nationwide open prospective registry on MSE (MULTIPOWER). The study was conducted according to the principle of the Declaration of Helsinki and was approved by the Ethics Committee of the coordinating hospital (Comité de Ética de la Investigación con medicamentos del Hospital Clínic de Barcelona) as well as by each Hospital Ethics Committee. All patients enrolled signed the informed consent. The article conforms to the STROBE guidelines for reporting observational studies.

### Study population

Inclusion criteria were: (1) age 18 or older; (2) indication for MSE according to local clinical practice; (3) informed consent signed. Exclusion criteria were: (1) known gastrointestinal varices; (2) severe and complicated eosinophilic esophagitis; (3) the presence of implantable devices in the intestinal tract; (4) pregnancy; (5) contraindications to general anaesthesia or deep sedation with propofol.

### MSE procedure

All procedures were performed with PowerSpiral, as described in a previous publication^5^. All endoscopists attended and passed a training course including a hands-on demonstration with MSE to get accreditation for its use.

Patients underwent MSE based on the findings' location from previous diagnostic exams, including capsule endoscopy, CT scan, or magnetic resonance enterography (MRE). In cases where previous diagnostic exams yielded negative results, but patients experienced SB bleeding, the anterograde route was chosen as the initial route of exploration, enabling a deeper or complete examination of the SB.

Total exploration was intended only in instances where: (1) the target lesion was not found at the suspected location, requiring a complete exploration to rule out the presence of the lesion, or (2) there was an inability to advance to the target lesion during the initial route access, requiring a combined bidirectional approach to complete the exploration.

All anterograde procedures were performed under general anaesthesia with oro- or nasotracheal intubation and patient monitoring (hemodynamic, ECG, and oxygen saturation monitoring). Retrograde procedures were performed under deep sedation with propofol, and the use of oxygen therapy administered via nasal cannula. Each procedure included the possibility of performing therapeutic interventions, such as polypectomy, hemoclipping and thermal coagulation, as well as the utilization of diagnostic adjuncts, including biopsies or India ink tattooing of lesions, where deemed necessary.

Patients who were on antiplatelet or anticoagulant therapy underwent management in accordance with the joint guidelines provided by the British Society of Gastroenterology and the European Society of Gastrointestinal Endoscopy (ESGE)^11^. This typically involves appropriate discontinuation and/or substitution of medications to safer alternatives, balancing the patient’s individual risk with the risk associated with the procedure.

### Variables

*Technical success*: The number or percentage of enteroscopies with the endoscope tip passing the angle of Treitz (anterograde route) or the ileocecal valve (retrograde route).

*Diagnostic yield*: The number or percentage of explorations with the detection of at least one lesion compatible with the indication for the exam.

*Therapeutic yield*: The number or percentage of enteroscopies with at least one therapeutic procedure, excluding biopsy.

*Interventional yield*: The number or percentage of explorations with at least one therapeutic procedure or biopsy.

Exploration times were defined as total exploration time (TET), from the beginning of the exploration to the withdrawal of the tip of the enteroscope; small bowel insertion time (SBIT) was calculated from the passage of the angle of Treitz or ileocecal valve to either the target lesion or maximum insertion point. Depth of maximum insertion (DMI) was determined from the angle of Treitz or ileocecal valve to either the target lesion or the furthest point of exploration. To calculate DMI, the number of SB folds encountered during withdrawal was counted, and this count was then converted into centimetres using the formula: cm = number of folds × 0.9^12,13^.

Abdominal surgeries encompassed any surgical procedure involving abdominal organs, excluding the abdominal wall, and were performed using either laparotomic or laparoscopic approach. These surgeries were further categorised into gastrointestinal (GI) surgeries, which directly or indirectly affected the distal oesophagus, stomach, small bowel, and colon, and other abdominal surgeries (all remaining cases). Unsuccessful enteroscopy was defined as a procedure not achieving diagnostic yield.

Experience with MSE at each hospital was evaluated according to the total number of procedures performed during the study period: apprentice centre ($\le$ 10 total procedures performed), experienced centre (between 11 and 50 procedures performed), and highly experienced centre (> 50 procedures performed).

Adverse events (AE) were registered from the beginning of anaesthesia or sedation to patient discharge from the hospital (pre-procedure and intra-procedure AEs). A follow-up phone call was made 14 days after the procedure to register post-procedure AEs. All AEs were reported according to the AGREE classification (Grade I to V), distinguishing between minor events (Grade I) and major events (Grade ≥ II)^14^.

### Endpoints

Primary endpoints were: (1) to describe the efficacy and safety of MSE according to prior abdominal surgeries; (2) to describe the efficacy and safety of MSE in patients with suspected SB strictures; (3) to describe the efficacy and safety of MSE in patients with prior unsuccessful balloon-enteroscopy.

Secondary endpoints were: (1) to identify predictors of efficacy (diagnostic yield) of MSE; (2) to describe MSE performance measures according to the endoscopist’s experience.

### Statistical analysis

The sample size calculation was based on the primary objective of the registry, which aimed to evaluate the efficacy of the technique (diagnostic yield) in both anterograde and retrograde explorations. At the onset of the study, only one clinical trial had been published, reporting a diagnostic yield of 74.2% in anterograde procedures, supplemented by another study indicating a yield of 71.4% (with 32% of patients undergoing retrograde procedures)^1,15^. Given these findings, our hypothesis was a global diagnostic yield of 70%. Utilizing a confidence level of 95% and a margin of error of 5%, we determined that a minimum of 323 explorations were required. All data were tested for normality with the Kolmogorov–Smirnov test and were non-normally distributed. Qualitative data were presented as number and percentage or 95% confidence interval. Quantitative data were presented as median and interquartile range (IQR). Univariate analysis was performed with the Chi-square test (or Fisher’s exact test) and Mann–Whitney U test when convenient. A multivariate binary logistic regression was performed to detect predictors of diagnostic yield, including all variables with *p* values < 0.1 at univariate analysis. The statistical significance level was set at 0.05 (two-sided). Statistical analysis was performed with SPSS Statistics (IBM Corp. Released 2019. IBM SPSS Statistics for Windows, Version 26.0. Armonk, NY: IBM Corp).

## Results

### Patient population

From June 2020 to July 2022 a total of 294 patients were enrolled. The median age was 65 years (IQR 51–75), and 55.1% (n = 162) were males. Median BMI was 25.4 (IQR 23–29.6) with 35% (n = 103) of patients in ASA III and 1.4% (n = 4) in ASA IV risk class. At least one prior abdominal surgery was present in 49% of patients (n = 144).

Globally, the number of enteroscopies performed was 326, with 81% of procedures performed in 3 Hospital centres. The route of exploration was oral in 66.6% of procedures (n = 217) and anal in the remaining 33.4% (n = 109); among these, there were 28 complete anterograde explorations and 11 combined explorations (oral + anal route). The total exploration rate was 12% (n = 39).

The overall technical success rate was 92.6% (n = 302), with a diagnostic yield of 66.3% (n = 216). Endoscopic findings during exploration included: angiodysplasias (n = 113), ulcers (n = 26), polypoid lesions (n = 21), subepithelial lesions (n = 10), ulcerated strictures (n = 13), fibrotic strictures (n = 10), Dieulafoy lesions (n = 7), diffused erosion areas (n = 6), ulcered neoplastic lesions (n = 5), diverticula (n = 5), and negative explorations (n = 86). The median SBIT was 37 min (IQR 25–55) and the DMI was 264 cm (IQR 115–423).

### Impact of prior abdominal surgeries on MSE

All enteroscopy procedures were divided according to prior gastrointestinal (GI) surgery (n = 44, 13.5%), other abdominal surgeries (n = 119, 36.5%), and no prior abdominal surgery (n = 163, 50%), see Table 1. The most common GI and abdominal surgeries were small bowel resection (43.2%) and hysterectomy (19.3%), respectively.

**Table 1:** Enteroscopy procedure data reported according to the type of abdominal surgery.

n	No surgery	Gastrointestinal surgery	Other abdominal surgery	*p*
	163	44	119	
Age, years (IQR)	65 (51–74)	63 (41–75)	67 (59–75)	0.053
BMI, Kg/m2 (IQR)	25 (23–29)	27 (22–31)	26 (24–30)	0.656
ASA class III-IV, n (%)	46 (28.2%)	11 (25%)	50 (42%)	0.025
Males, n (%)	103 (63.2)	22 (50)	51 (42.9)	0.003
Oral route, n (%)	112 (68.7)	23 (52.3)	82 (68.9)	0.097
Technical success, % (95% CI)	93.3 (88.2–96.6)	90.9 (78.3–97.5)	92.4 (86.1–96.5)	0.865
Diagnostic yield, % (95% CI)	67.5 (59.7–74.6)	47.7 (32.5–63.3)	71.4 (62.4–79.3)	0.016
Interventional yield, % (95% CI)	71.8 (64.2–78.5)	56.8 (41–71.7)	75.6 (66.9–83)	0.061
SBIT, min (IQR)	34 (24–60)	37 (27–56)	41 (28–55)	0.642
TET, min (IQR)	58 (43–84)	57 (37–80)	61 (45–84)	0.567
DMI, cm (IQR)	275 (117–426)	162 (63–315)	303 (146–450)	0.037
AE, % (95%CI)	10.4 (6.2–16.2)	11.4 (3.8–24.6)	10.9 (5.9–18)	0.845
Total enteroscopy rate, % (n)	13.5 (22)	4.5 (2)	12.6 (15)	0.258

*AE* adverse event, *ASA* American Society of Anesthesiology, *BMI* body mass index, *DMI* depth of maximum insertion, *SBIT* small bowel insertion time, *TET* total exploration time.

Among the three groups no significant differences were observed as per route of exploration (*p* = 0.097), technical success (*p* = 0.865), small bowel insertion time (*p* = 0.642), and TET (*p* = 0.567), however, in comparison with patients with other abdominal surgery and no prior surgery, patients with GI surgery presented a significantly lower diagnostic yield (47.7% vs 71.4 and 71.8%, *p* < 0.001), together with a lower DMI (162 cm vs 303 vs 275, *p* = 0.037).

### Efficacy of MSE after prior unsuccessful balloon enteroscopy

**Figure 1:**
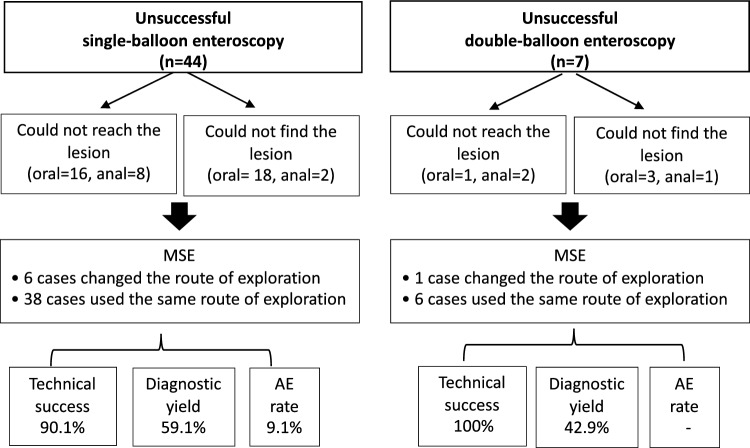
Flaw-chart of MSE exploration in patients with prior unsuccessful balloon-enteroscopy.

In the present cohort, 51 enteroscopy procedures were performed after unsuccessful balloon enteroscopy (7 procedures with double-balloon enteroscopy and 44 with single-balloon enteroscopy), see Fig. 1. All balloon-enteroscopies were performed in highly experienced centres. Within this subgroup of procedures, MSE showed a technical success of 92.2% (n = 47) and diagnostic yield of 56.9% (n = 29), with a total AE rate of 7.8% (n = 4) and no major AEs reported.

### MSE in small bowel strictures

In 49 procedures the indication for enteroscopy was a suspected small bowel stricture, detected by capsule endoscopy (n = 12), CT scan or MRI-enterography (n = 37). The full description is presented in Table 2. Globally, the diagnostic yield was 59.2% (n = 29), with an interventional yield of 71.4% (n = 35) and a total AE rate of 8.2% (n = 4). A fibrotic or ulcered intestinal stricture was confirmed in 46.9% of cases (n = 23), and in 14.3% of cases (n = 7) an endoscopic CRE (Controlled Radial Expansion) balloon dilation was performed. Conversely, in 7 cases (14.3%), the SB could not be explored due to intestinal fixation (technical unsuccess). No SB strictures were overpassed by the spiral overtube. No major AEs were observed.

**Table 2:** Indication of MSE exploration in patients with suspected SB strictures.

Clinical presentation	Pre-enteroscopy diagnostic procedure	Stricture location	Route of enteroscopy exploration	MSE Technical success	MSE Diagnostic yield	Stricture confirmed	Intervention	Total AE
Suspected IBD, chronic diarrhea with inflammatory biomarkers (n = 13)	CE (3) CT scan (2) MRI-enterography (8)	Proximal jejunum (3) Distal jejunum (2) Proximal ileum (2) Distal ileum (6)	Oral (7) Anal (6)	100% (13/13)	69.2% (9/13)	46.2% (6/13)	Endoscopic balloon dilation (3) APC (1) Biopsy (9)	Abdominal pain (1)
Chronic anemia (n = 9)	CE (8) CT scan (1)	Proximal jejunum (1) Distal jejunum (2) Proximal ileum (2) Distal ileum (4)	Oral (5) Anal (4)	88.8% (8/9)	77.7% (7/9)	66.6% (6/9)	Endoscopic balloon dilation (2) Biopsy (7)	–
Abdominal pain (n = 23)	CT scan (10) MRI-enterography (12) CE (1)	Proximal jejunum (1) Distal jejunum (3) Proximal ileum (4) Distal ileum (15)	Oral (8) Anal (15)	82.6% (19/23)	52.2% (12/23)	47.8% (11/23)	Capsule Endoscopy extraction (1) Endoscopic balloon dilation (2) Biopsy (16)	Abdominal pain (2) Difficult withdrawal through the esophagus (1)
Incidental finding (n = 4)	CT scan (4)	Proximal jejunum (1) Distal jejunum (1) Proximal ileum (0) Distal ileum (2)	Oral (3) Anal (1)	50% (2/4)	25% (n = 1)	–	–	–

*AE* adverse events, *CE* capsule endoscopy; *CT* coaxial tomography, *IBD* inflammatory bowel disease; *MRI* magnetic resonance imaging.

### Predictors of diagnostic yield

At univariate analysis, TET (*p* = 0.055), prior GI surgery, indication for SB exploration, and route of exploration were significant factors related to diagnostic yield (*p* < 0.1). At multivariate analysis, prior GI surgery was the only factor negatively associated with diagnostic yield (OR 0.128, *p* = 0.004). Data are presented in Table 3.

**Table 3:** Univariate and multivariate analysis of factors associated with MSE diagnostic yield.

	Univariate			Multivariate			
	Dyield (Yes)	Dyield (No)	*p*	OR	95%CI		*p*
Sex (male), n (%)	118 (54.6%)	58 (52.7%)	0.745				
Age, years (IQR)	67 (60–75)	64 (50–77)	0.363				
BMI, Kg/m^2^ (IQR)	26 (24–30)	26 (23–31)	0.832				
SBIT, min (IQR)	41 (28–68)	40 (25–58)	0.684				
TET, min (IQR)	65 (49–92)	60 (41–82)	0.055	1.007	0.981	1.035	0.589
DMI, cm (IQR)	252 (122–414)	257 (73–410)	0.190				
Prior GI surgery, n (%)	21 (9.7%)	23 (20.9%)	0.005	0.128	0.032	0,509	0.004
ASA Class III/IV, n (%)	74 (34.4%)	33 (30%)	0.439				
Oral route, n (%)	151 (69.9%)	66 (60%)	0.073	0.983	0.297	3.254	0.977
Indication (SB bleeding), n (%)	157 (72.7%)	61 (55.5%)	0.001	0.899	0.739	1.092	0.282

*ASA* American Society of Anesthesiology, *BMI* body mass index, *DMI* depth of maximum insertion, *GI* gastrointestinal, *IQR* interquartile range, *SBIT* small bowel insertion time, *TET* total exploration time.

### Efficacy and safety according to endoscopist’s experience

**Figure 2:**
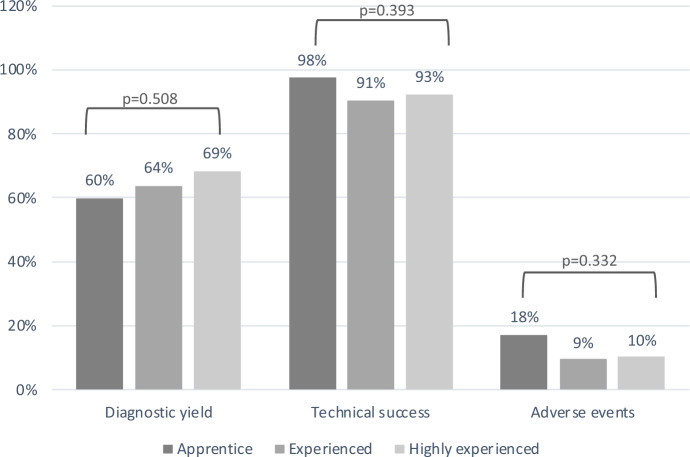
Comparison of MSE performance according to endoscopist’s experience.

All procedures were analyzed according to the level of experience of the endoscopist (apprentice, experienced, highly experienced). No significant differences were detected among groups as per technical success (*p* = 0.393), diagnostic yield (*p* = 0.508), and adverse events (*p* = 0.332), see Fig. 2.

### MSE safety: early and late adverse events

The total AE rate was 10.7% (n = 35). The majority of AEs were classified as AGREE grade I, including self-limited post-procedure abdominal pain (4.0%, n = 13), difficult withdrawal through the oesophagus without sequelae (2.8%, n = 9), diarrhoea (0.3%, n = 1), cervical pain (0.3%, n = 1), fever (6.1%, n = 2), vomiting (0.3%, n = 1), bradycardia (0.3%, n = 1), and bronchospasm (0.3%, n = 1). The rate of major AEs (AGREE Grade ≥ II) was 1.8% (n = 6), as detailed in Table  4. Asymptomatic superficial mucosal erythema with erosions of the small bowel and the oesophagus were observed in most of the procedures upon withdrawal and related to the spiral technique itself. These were not classified as AEs.

**Table 4:** Description of major adverse events according to AGREE classification.

AGREE classification	Adverse event description	Patients (n)	Related to MSE	Follow-up
Grade II	Withdrawal issues During anterograde MSE, difficult and prolonged withdrawal of the enteroscope was experienced at mid-proximal esophagus, without significant esophageal lesions or therapeutic intervention, but requiring 24–36 h of hospital admission	4	Yes	No sequelae
	Self-limited Intestinal hemorrhage A patient with mitral insufficiency and atrial fibrillation on acenocoumarol underwent anterograde MSE. A 20 mm polyp was detected and treated with epinephrine injection, hot snare polypectomy, and hemostatic clip placement. No immediate complications. After 48 h of anticoagulation reintroduction, patient had self-limited melena and was observed for 48 h at the hospital without further intervention	1	No	No sequelae
Grade IIIb	Jejunal acute ischemia An 80-year-old man presented with a 10 mm angiodysplasia in the jejunum detected by CE. The lesion was identified through MSE and treated with APC, epinephrine and polidocanol due to persistent bleeding. Ultimately, two hemostatic clips were placed. Patient was discharged asymptomatic At 48 h following MSE, the patient experienced acute abdominal pain along with leukocytosis. A CT scan revealed acute jejunal ischemia, leading to the confirmation of transmural ischemic necrosis through jejunal resection	1	No	No sequelae

*APC* Argon plasma coagulation; *CE* Capsule endoscopy; *MSE* Motorized spiral enteroscopy; *CT* Computerized tomography.

Early AEs (< 24 h from exploration beginning) were 29 (8.9%), of which 16 occurred during the exploration. Delayed AEs (24 h to 14 days from the procedure) were 6 (1.8%). No differences were detected in relation to prior abdominal surgery (*p* = 0.845) or indication of suspected SB strictures (*p* = 0.896). The total AE rate in therapeutic and non-therapeutic procedures was 11.3% and 10.2%, respectively (*p* = 0.748). No differences were observed related to the oral or anal route of exploration (11.1 and 10.1%, *p* = 0.790).

## Discussion

The present multicenter study aims to investigate the efficacy and safety of MSE in patients with prior abdominal surgery, SB strictures, or unsuccessful enteroscopy to identify the optimal target population for this novel technique.

MSE has been recognized as a highly efficient technique with notable diagnostic yield, insertion depth and short exploration times^16,17^. A recent meta-analysis based on nine clinical studies reported a pooled diagnostic yield of 78%, with a technical success of 98% and 97% for anterograde and retrograde routes, respectively^18^. However, these findings were based on data with a low percentage of patients with prior abdominal surgery, with a focus on anterograde explorations.

Early clinical trials excluded patients with prior abdominal surgeries due to safety reasons^1,4^. However, recent studies that included such patients have shown no specific safety issues, although the sample sizes were limited and the definition of abdominal surgery varied^3,5,6^. In the present study, 49% of patients had at least one prior abdominal surgery. Based on previously described experiences suggesting a different impact in relation to the type of abdominal surgery, the patient population was divided into those with prior gastrointestinal surgery, directly implicating the gut and those with other abdominal surgeries^5^. The results indicate that gastrointestinal surgery may negatively affect the exploration, as this group of patients compared to those with no surgery, showed a lower diagnostic yield (47.7 vs 67.5%), and a lower DMI (162 vs 275 cm), indicating a more challenging progression of the enteroscope which would probably limit the detection of suspected lesions. Moreover, prior gastrointestinal surgery was the only independent predictor of diagnostic yield in multivariate analysis. Interestingly, patients with other abdominal surgeries showed similar or even better performance compared to those with no history of surgery, in terms of diagnostic yield (71.4 vs 67.5%) and DMI (303 vs 275 cm). These findings suggest that abdominal surgeries not involving the gut do not significantly halt the progression of the endoscope.

Similarly, another exclusion criterion in early clinical trials was SB strictures^1,3^. The passage of the spiral overtube, with its 18 mm diameter during MSE could unintentionally cause dilation of the stricture, leading to intestinal lacerations or perforation. However, in this clinical scenario, enteroscopy can still be valuable for reaching the pre-detected stricture, evaluating its characteristics, obtaining biopsies, performing endoscopic dilation, or tattooing the area for possible subsequent intervention. In the present cohort, strictures were confirmed in 46.9% of procedures with high interventional yield (71.4%) including 14.3% of endoscopic CRE balloon dilation. The passage of strictures with the endoscope was intentionally avoided under any circumstances, and no major AEs were associated with this approach. This is the first study to our knowledge to demonstrate the effectiveness and safety of exploring patients with suspected small bowel strictures without attempting to pass them with the spiral overtube.

The present study also includes patients with prior unsuccessful balloon enteroscopy (n = 51), due to lack of technical success or failed diagnostic yield. In this specific patient cohort, MSE achieved a technical success rate of 92.2% (n = 47), a diagnostic yield of 56.9% (n = 29), and a total AE rate of 7.8% (n = 4) with no major AEs. These findings suggest that MSE may be a viable option for a second attempt exploration, likely due to its ability to navigate difficult anatomy more easily (improved technical success) and thoroughly explore the small bowel (high insertion depth).

On the other hand, safety is a major concern regarding MSE^19,20^. Recent evidence suggests that the majority of AEs are minor and seem to be associated with the small bowel endoscopy procedure itself, rather than the specific technique of MSE^16–18^. However, it is essential to acknowledge that certain AESs specifically related to MSE have occurred, potentially leading to severe complications^20^. The utilization of a relatively flexible 18 mm overtube during MSE has exposed the technique to a higher risk of mucosal laceration and subsequent perforation, along with difficulties in withdrawal and the inability to intervene in case of unexpected complications^22–24^.

The present study examined various clinical scenarios that could pose a higher risk to patients. The overall major AE rate was 1.8%, which is below the 5% threshold recommended by ESGE quality measures^21^. Prior gastrointestinal surgery did not increase adverse event rates compared to non-operated patients or those with other abdominal surgeries (11.4 vs 10.4 vs 10.9, *p* = 0.845). The most common major AE encountered was the challenging withdrawal of the enteroscope through the oesophagus, observed in four anterograde explorations. This led to a prolonged exploration time and required one day of hospital observation. The withdrawal of MSE is accomplished by an anticlockwise rotation of the spiral overtube, which helps release the folded intestine. The most challenging area for withdrawal was typically the mid-proximal oesophagus, with the tip of the enteroscope at the level of the stomach. It can be hypothesized that a pronounced anatomical curve in the cervicothoracic oesophagus or the spasm of the lower oesophagal sphincter may trigger the activation of the enteroscope's security system, causing the spiral rotation to stop and hinder the passage of the endoscope. In the four cases described, the enteroscope was eventually extracted by repeated forward and backward movements while adjusting the position of the neck.

Finally, in the present study, performance results were compared based on the level of experience with MSE. Since the first feasibility trial, it has been postulated that proficiency in the MSE technique can be achieved after performing approximately 10 procedures^1^. Endoscopists were categorized as apprentice, experienced, and highly experienced, and no significant differences were found among the three groups in terms of technical success, diagnostic yield, and adverse events. However, it should be noted that a major limitation of these results is that all endoscopists involved in the study came from tertiary referral hospitals and had prior experience in balloon enteroscopy. Based on our data, a hands-on course and the completion of 10 procedures appear to be sufficient training for endoscopists with prior experience in balloon enteroscopy.

The present study has certain limitations that merit acknowledgment.

Firstly, its reliance on registry data imposes constraints on the depth of analysis possible for variables related to efficacy and safety. Specifically, the primary focus of the registry was to assess the diagnostic yield, meaning it lacks sufficient power to comprehensively address other objectives through sub-analyses. Nonetheless, it is worth noting that the study stands out as one of the most extensive multicentre experiences on MSE, and its design is prospective.

Secondly, the registry was specifically designed to observe MSE technique, thereby preventing a direct comparison with other techniques. The analysis of failure from balloon-enteroscopy in the study relies on partial retrospective data, and the relatively low sample size prevents from a comprehensive analysis of the transition from failed balloon-enteroscopy to MSE. Consequently, it does not allow to define a superiority over other techniques; instead, it merely suggests MSE as a potential add-on therapy or alternative option.

Finally, the result of GI surgery impact on MSE is intriguing and is probably related to the effect of adhesions and surgical fixation. Exploring the impact of surgical access (open vs. laparoscopic) could help elucidate the true causal factors behind such difficulties. However, the limited sample size in this specific analysis precluded such an investigation.

In summary, MSE is a valid technique for small bowel exploration, but less efficient in patients with prior intestinal surgery. MSE can assess and treat SB strictures, but caution is advised to avoid complications. MSE is effective after failed previous balloon enteroscopy, but more research is required. AE rates are similar to other techniques and not influenced by the clinical scenario.

Although published evidence from single-center and multicenter cohorts, along with present data, described an AE rate consistent with that observed in small bowel endoscopy, due to general safety concerns, the manufacturer of PowerSpiral decided to permanently withdraw the enteroscope in July 2023^25^. This irrevocable decision has paved the way for new avenues in small bowel interventional endoscopy. Presently, the most widely adopted systems are balloon-assisted, despite known limitations such as prolonged procedure times. Several proposals have been suggested to enhance the safety and efficacy of MSE. In the opinion of the authors of this article, restricting its application to the retrograde approach to mitigate technique-related adverse events or confining its usage to highly experienced centers could be beneficial. Furthermore, adjustments to the materials and system of spiral enteroscopy could facilitate swift withdrawal during emergency scenarios. The present study sheds light on both the benefits and limitations of MSE, thereby contributing to the ongoing process of refining this technique and exploring future solutions. Our findings underscore the importance of continuous improvement in MSE, highlighting the existing unmet needs for balloon enteroscopy techniques.

## Data Availability

The datasets generated during and/or analysed during the current study are derived from the MULTIPOWER nationwide registry and are not publicly available due to data protection and privacy legislation but are available from the corresponding author on reasonable request.

## References

[1] Beyna, T. *et al.* Motorised spiral enteroscopy: First prospective clinical feasibility study. *Gut* **70**, 261–267 (2021). 32332141 10.1136/gutjnl-2019-319908

[2] Beyna, T. *et al.* Total motorized spiral enteroscopy: First prospective clinical feasibility trial. *Gastrointest. Endosc.*10.1016/j.gie.2020.10.028 (2021). 33144239 10.1016/j.gie.2020.10.028

[3] Beyna, T. *et al.* Motorized spiral enteroscopy: Results of an international multicenter prospective observational clinical study in patients with normal and altered gastrointestinal anatomy. *Endoscopy* **54**, 1147–1155 (2022). 35451040 10.1055/a-1831-6215

[4] Ramchandani, M. *et al.* Diagnostic yield and therapeutic impact of novel motorized spiral enteroscopy in small-bowel disorders: A single-center, real-world experience from a tertiary care hospital (with video). *Gastrointest. Endosc.* **93**, 616–626 (2020). 32663489 10.1016/j.gie.2020.07.001

[5] Giordano, A. *et al.* Motorized spiral enteroscopy is effective in patients with prior abdominal surgery. *Dig. Dis. Sci.* **68**, 1447–1454 (2023). 36104534 10.1007/s10620-022-07688-1 PMC10102144

[6] Al-Toma, A. *et al.* The performance and safety of motorized spiral enteroscopy, including in patients with surgically altered gastrointestinal anatomy: A multicenter prospective study. *Endoscopy* **54**, 1034–1042 (2022). 35226945 10.1055/a-1783-4802

[7] Pal, P. *et al.* Diagnostic yield and technical performance of the novel motorized spiral enteroscopy compared with single-balloon enteroscopy in suspected Crohn’s disease: A prospective study (with video). *Gastrointest. Endosc.* **97**, 493–506 (2023). 36273601 10.1016/j.gie.2022.10.017

[8] Chan, W. *et al.* Motorized spiral enteroscopy versus double-balloon enteroscopy: A case-matched study. *Gastrointest. Endosc.* **97**, 314–324 (2023). 36228697 10.1016/j.gie.2022.09.030

[9] Pal, P. *et al.* Technical performance and diagnostic yield of motorised spiral enteroscopy compared with single-balloon enteroscopy in suspected Crohn’s disease: A randomised controlled, open-label study (the MOTOR-CD trial). *Gut* **72**, 1866–1874 (2023). 37197904 10.1136/gutjnl-2023-329885

[10] Rughwani, H. *et al.* A Randomized, controlled trial comparing the total enteroscopy rate and diagnostic efficacy of novel motorized spiral enteroscopy and single-balloon enteroscopy in patients with small-bowel disorders: The motor trial (NCT 05548140). *Am J Gastroenterol* **118**, (2023). 10.14309/ajg.0000000000002409 37463435

[11] Veitch, A. M. *et al.* Endoscopy in patients on antiplatelet or anticoagulant therapy: British Society of Gastroenterology (BSG) and European Society of Gastrointestinal Endoscopy (ESGE) guideline update. *Gut* **70**, 1611–1628 (2021). 34362780 10.1136/gutjnl-2021-325184 PMC8355884

[12] Efthymiou, M. *et al.* SINGLE-01: A randomized, controlled trial comparing the efficacy and depth of insertion of single- and double-balloon enteroscopy by using a novel method to determine insertion depth. *Gastrointest. Endosc.* **76**, 972–980 (2012). 22980289 10.1016/j.gie.2012.06.033

[13] Akerman, P. A., Agrawal, D., Cantero, D. & Pangtay, J. Spiral enteroscopy with the new DSB overtube: A novel technique for deep peroral small-bowel intubation. *Endoscopy* **40**, 974–978 (2008). 19065477 10.1055/s-0028-1103402

[14] Nass, K. J. *et al.* Novel classification for adverse events in GI endoscopy: The AGREE classification. *Gastrointest. Endosc.* **95**, 1078-1085.e8 (2022). 34890695 10.1016/j.gie.2021.11.038

[15] Giordano, A. *et al.* Preliminary experience with the novel motorized spiral enteroscope (powerspiral) for small bowel diseases. *Endoscopy* **52**, OP121 (2020).

[16] Wang, Y., Ma, B., Li, W. & Li, P. Effectiveness and safety of novel motorized spiral enteroscopy: A systematic review and meta-analysis. *Surg. Endosc.* **37**, 6998–7011 (2023). 37349592 10.1007/s00464-023-10179-7

[17] Nabi, Z. *et al.* Role of novel motorized enteroscopy in the evaluation of small bowel diseases: A systematic review and meta-analysis. *J. Clin. Gastroenterol.* **58**, 349–359 (2024). 37279281 10.1097/MCG.0000000000001862

[18] Papaefthymiou, A. *et al.* Performance and safety of motorized spiral enteroscopy: A systematic review and meta-analysis. *Gastrointest. Endosc.* **97**, 849–858 (2023). 36738795 10.1016/j.gie.2023.01.048

[19] Pennazio, M. Safety of motorized spiral enteroscopy: Is what we know today enough?. *Endoscopy* **54**, 1156–1157 (2022). 35858637 10.1055/a-1881-4691

[20] Yamamoto, H., Despott, E. J., González-Suárez, B., Pennazio, M. & Mönkemüller, K. The evolving role of device-assisted enteroscopy: The state of the art as of August 2023. *Best Pract. Res. Clin. Gastroenterol.* **64–65**, 101858 (2023). 37652651 10.1016/j.bpg.2023.101858

[21] Spada, C. *et al.* Performance measures for small-bowel endoscopy: A European Society of Gastrointestinal Endoscopy (ESGE) quality improvement initiative. *Endoscopy* **51**, 574–598 (2019). 31075800 10.1055/a-0889-9586

[22] Rajput, S. *et al.* Spontaneous migration of spiral fin overtube during motorized spiral enteroscopy. *ACG Case Rep. J.* **9**, e00808 (2022). 35784503 10.14309/crj.0000000000000808 PMC9246071

[23] Wiedbrauck, D. *et al.* Spontaneous detachment of the spiral overtube from the endoscope in the upper esophagus: A case report and literature review of a rare complication during motorized spiral enteroscopy. *Z. Gastroenterol.* **62**, 407–411 (2024). 37044124 10.1055/a-2053-9238

[24] Pal, P., Rebala, P., Nabi, Z., Tandan, M. & Reddy, D. N. Small-bowel transection after peroral motorized spiral enteroscopy. *iGIE* **2**, 271–272 (2023). 10.1016/j.igie.2023.05.004 PMC12850818 41646992

[25] Olympus Medical. Urgent field safety notice QIL FY24-EMEA-10-FY24-OMSC-05 2023 [urgent field safety notice QIL FY24-EMEA-10-FY24-OMSC-05]. https://www.igj.nl/binaries/igj/documenten/waarschuwingen/2023/07/11/fsn-qil-fy24-emea-10-fy24-omsc-05-olympus-medical-systems-corporation-powerspiral-intestinal-videoscope-psf-1/IT2081382+FSN-QIL+FY24-EMEA-10-FY24-OMSC-05+Olympus+Medical+Systems+Corporation+PowerSpiral+Intestinal+Videoscope+PSF-1.pdf

